# Reported severity of psychotic, depressive and anxiety symptoms in relation to bilingual language profile: An exploratory study and the validation of Basque versions of the PQ-B, DASS-42, PHQ-9 and GAD-7

**DOI:** 10.1371/journal.pone.0314069

**Published:** 2025-03-03

**Authors:** Leire Erkoreka, Naiara Ozamiz-Etxebarria, Onintze Ruiz, Maider Prieto, Saioa Aspiazu, Argiñe Mingo, Urko Aguirre, Miren Orive, Simona Mancini

**Affiliations:** 1 Galdakao-Usansolo University Hospital, Osakidetza Basque Health Service, Galdakao, Spain; 2 University of the Basque Country UPV-EHU, Leioa, Spain; 3 BioBizkaia Health Research Institute, Barakaldo, Spain; 4 CIBERSAM ISCII, Madrid, Spain; 5 Bizkaia Mental Health Network, Osakidetza Basque Health Service, Bilbao, Spain; 6 Red de Investigación en Cronicidad, Atención Primaria y Promoción de La Salud (RICAPPS), Galdakao, Spain; 7 Basque Center on Brain, Language and Cognition, Donostia-San Sebastian, Spain; 8 Ikerbasque, Basque Foundation for Science, Bilbao, Spain; Nanyang Technological University, SINGAPORE

## Abstract

**Background:**

Language plays a crucial role in health care and especially in mental health, since the use of the native language helps to make a good diagnosis as several studies have shown.

**Aim:**

We studied the influence of language on the accurate detection of psychotic and affective symptoms, exploring differences in the severity of reported symptomatology in a bilingual Basque-Spanish population.

**Methods:**

The study uses the Prodromal Questionnaire-Brief for the detection of psychosis and the Patient Health Questionnaire-9, Generalized Anxiety Disorder Scale-7, and Depression, Anxiety and Stress Scale-42 for the assessment of stress, anxiety and depression. Basque versions of the scales were developed and their psychometric properties were evaluated in a sample of 623 individuals, including 521 from the general population and 102 psychiatric patients. Possible relations between questionnaire scores and four linguistic factors, namely first language (L1), proficiency, age of acquisition and language exposure, were examined.

**Results:**

The four translated questionnaires showed adequate sensitivity, goodness-of-fit, and reliability indices, thus validating their suitability for general and clinical settings. The results showed that reporting of depressive symptoms seemed to be modulated by linguistic variables, mainly L1, whereas the severity of psychotic symptoms was less reliably associated with the gathered linguistic factors.

**Conclusions:**

Overall, our results suggest that language of assessment by means of written instruments may have a limited impact on healthcare outcomes in balanced bilingual populations. The study enriches the understanding by considering various linguistic factors beyond L1, and by exploring the effect of these factors on affective symptoms, apart from psychotic ones.

## 1. Introduction

### 1.1 Language, psychiatric evaluation and assessment tools

Language is a core element of healthcare that notably impacts the interaction between the clinician and the patient particularly when it is not the patient´s first language. As many authors have pointed out, when assessing the patient’s symptomatology, it is important that the patient understands the language being used by the clinician in order to avoid errors and misunderstandings [1], as well as to improve adherence [2] and accessibility to mental health services [3]. The patient’s experience, which is increasingly considered as important over the last years, is also influenced by language barriers [4].

The lack of measurable physical signs that function as biomarkers of psychiatric conditions means that diagnostic assessment and subsequent follow-up depend primarily on language-based clinician-patient interaction. The use of standardized structured interviews as well as psychometric scales is currently the best way to make objective assessments that can be universally reliable. To achieve these universal standards, the language variable comes into play again, since it is essential to adapt these instruments, not only linguistically but also culturally to different countries and sociolinguistic environments.

In the field of mental health, and regarding psychotic symptomatology in particular, our group has already reported a meta-analysis that revealed that the language used in the evaluation of bi- and multilingual patients makes a difference in how accurately psychotic symptoms are detected [5]. Specifically, psychotic symptoms may be more severely rated when the clinical assessment is conducted in the first language (L1) of the patient rather than in the second language (L2). A previous investigation [6] and published case reports point in the same direction [7–12]. Less is known about possible differences in the detection of affective symptoms in balanced bilingual patients when assessing them in L1 *vs*. L2.

The available literature indicates that autobiographical memories seem to be more emotionally expressive in the L1 of bilingual individuals, and that the communication of memories of personal events is qualitatively different in the two languages [13]. An emotional barrier and increased defensiveness have been detected when a bilingual patient uses the L2 and more affect is observed when patients express themselves in L1 [14,15]. Other authors claim that affect is weaker in L2 and information processing less automatic, which reduces emotionality [16]. Emotional distance in L2 has also been shown to affect even automatic stages of emotional processing [17].

Among English-Welsh bilingual patients, a differential experience of languages in auditory hallucinations has been reported, influenced by the Age of Acquisition (AoA) of each language, as well as the frequency of use and the proficiency in each language [18]. Linguistic differences in auditory hallucinations had already been detected in earlier studies [9,19], including our Spanish-Basque bilingual setting. Also, a case study describing the encapsulation or decrease of psychotic symptoms when using the second language has recently been published [20].

Most of previous research has been conducted using semi-structured interviews or non-standardized clinical assessments. Thus, whether these differences are also observed when using written assessments, such as psychometric tools, remains unknown.

### 1.2 Validation of psychometric tools

Psychometric tools are widely used for the assessment of neurocognitive functioning, evaluating personality traits, quantifying psychiatric symptoms, estimation of adaptability to the environment and also for screening purposes. However, most of these tools have been created in English. In order to use these tools with diverse populations, they must be translated and then validated through administration to groups within the target population. There is a broad consensus about the methods to ensure rigorous translation and validation across different cultural settings, and in fact, the utility of most existing psychometric tools relies on translations into different languages and their validation for distinct cultural environments [21,22].

#### Detection of prodromal psychosis with the Prodromal Questionnaire-Brief

Early detection and intervention in psychosis has the potential to improve the prognosis of the condition [23–25] and thus, the identification of individuals at clinical high risk for psychosis has become an extensive focus of research and debate in the last two decades. Semi-structured interviews such as the Structured Interview for Psychosis-risk Syndromes or the Comprehensive Assessment of At-risk Mental States have enabled the use of common criteria by researchers embarking on investigation in this field. Nonetheless, these measures require referrals to specialized programs and so are not suitable for screening purposes in large cohorts. Thus, more recently, self-report instruments have been developed as a means to screen larger populations, in order to facilitate the detection of a much greater number of high-risk individuals.

Among the available self-report instruments, the Prodromal Questionnaire-Brief (PQ-B) has demonstrated good convergence with validated interview measures, such as the previously mentioned Structured Interview for Psychosis-risk Syndromes and the Comprehensive Assessment of At-risk Mental States [26–30]. The PQ-B has also shown excellent sensitivity to detect emerging psychosis and a strong agreement with clinician-rated attenuated psychotic symptoms over a six-month follow-up study [31].

#### Stress, anxiety and depression screening with Patient Health Questionnaire-9, GAD-7 and Depression, Anxiety and Stress Scale-42

Depressive and anxiety disorders are a major contributor to the global burden of disease [32]. Therefore, there is a clear need for reliable tools to evaluate and identify patients with potential affective disorders. These high incidences have motivated efforts to improve the detection and management of depression and anxiety by routinely administering screeners. One of the most widely‐used self‐report depression screeners is the Patient Health Questionnaire-9 [33,34]. As far as anxiety disorders are concerned [35], GAD Scale-7 (GAD-7) was developed to identify probable cases of GAD and to assess symptom severity [36]. Finally, Depression, Anxiety and Stress Scale (DASS-42, [37] is another self-report measure which has the advantage of exploring depression, anxiety and stress in a single instrument, that is extensively used both in research and in clinical practice.

### 1.3. The present study

The present study is conducted in a bilingual region in which the Basque and Spanish languages coexist. The translation and validation of patient-related outcome measures is part of a series of initiatives that have been undertaken over the last few years within the Basque public health system to ensure patients can choose the language they wish to be assisted in. Self-reports that evaluate psychiatric symptoms are among these tools.

In this context, we used the validation process of the Basque versions of the PQ-B, the PHQ-9, the GAD-7 and the DASS-42 scales to explore whether differences in reported symptomatic severity would be detected when the language of assessment (Basque) was the L1 or the L2 of the informant. We also assessed whether there were independent effects of AoA, proficiency and exposure to Basque on the reporting of symptoms. To that end, participants in the study were asked a series of questions concerning their linguistic profile (see Supplementary Materials 8). Considering that most of the world’s population is bi- or multilingual, the results will be of great interest in terms of public health policies, insofar as they will help to clarify whether linguistic choice has clinical relevance in terms of the accurate detection of psychiatric symptoms.

Therefore, the aim of the present study is twofold. On the one hand, we developed the Basque versions of the PQ-B, the PHQ-9, the GAD-7 and the DASS-42, and tested their psychometric properties. On the other hand, we investigated whether any differences exist in the reported severity of psychopathology according to linguistic variables in a large sample of bilingual individuals from both general and clinical populations.

When the language of assessment is Basque, it is expected that higher symptomatic severity will be reported by L1 than L2 Basque speakers. It is also expected that higher proficiency and exposure to Basque and earlier AoA will be associated with more accurate detection of symptoms.

## 2. Methods

### 2.1. Participants and procedures

A total of 623 Basque-Spanish bilingual individuals were recruited for the study, 521 were healthy individuals from the general population and 102 were psychiatric patients from different clinical settings. However, 15 participants were excluded due to lack of linguistic profile and demographic information, leaving 512 participants in the general group and 96 participants in the clinical group. Participants from the general population were mainly students and employees of the University of the Basque Country UPV/EHU, volunteers recruited from the Basque Center on Cognition, Brain and Language (BCBL) database, and spontaneous volunteers from the general population that found out about the project through informal verbal diffusion. Participants from the clinical settings were heterogeneous in- and outpatients recruited at hospital wards and community mental health centers of the Basque public health system.

**Fig 1:**
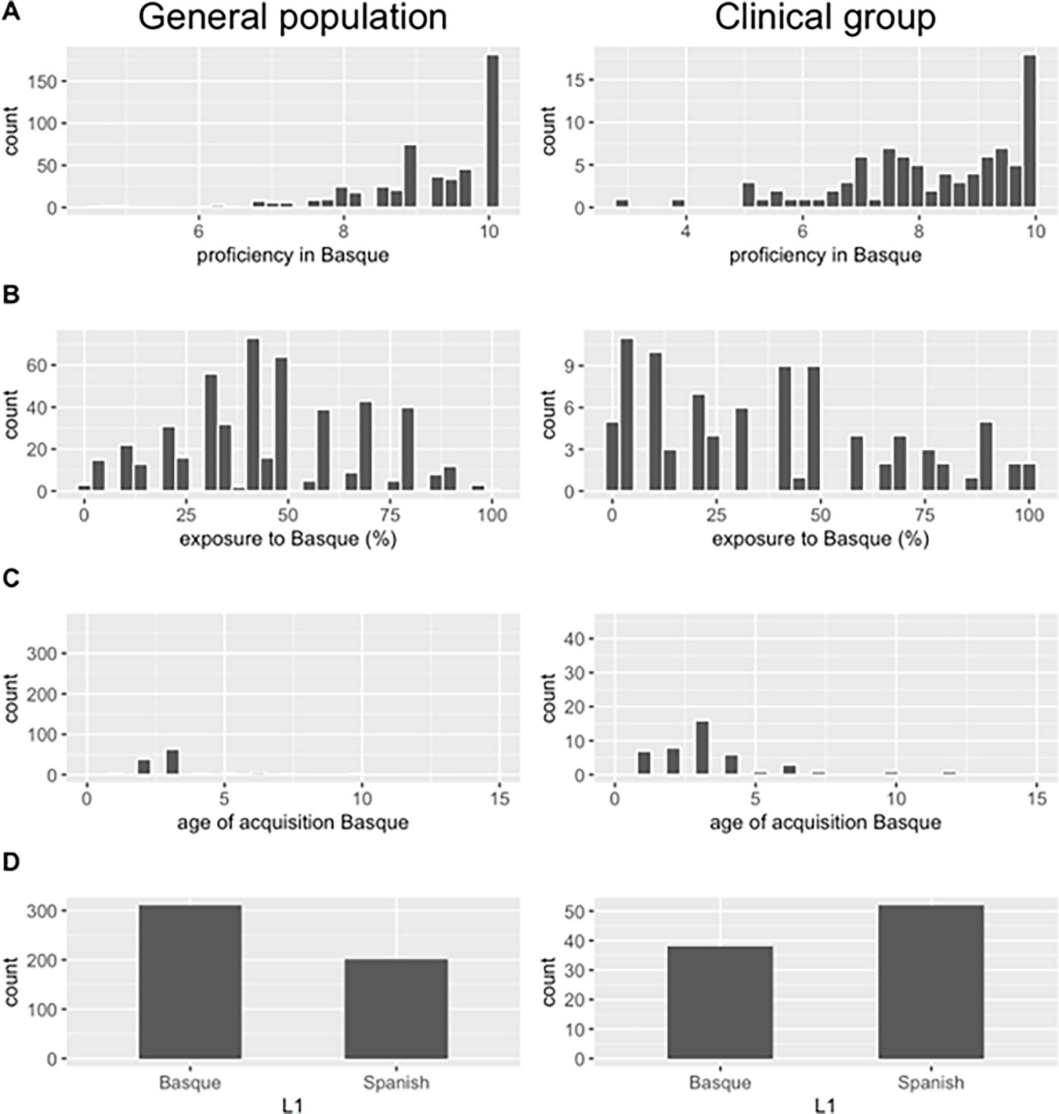
Linguistic profile of the overall general (N = 521) and clinical (N = 102) populations tested.

Socio-demographic data including age, gender, marital status, level of education and employment were collected in an ad-hoc questionnaire. Next, participants completed a language competence questionnaire developed by the BCBL. Afterwards, they completed the Basque version of the PQ-B, the Basque version of PHQ-9, the Basque version of GAD-7 and the Basque version of the 42-item Depression, Anxiety and Stress Scale (DASS-42). All the mental health screeners had been previously translated using the back-translation method, and their back-translated English version had received the approval of the respective authors (see Section 2.3). The questionnaires were administered to the general population group by means of an online platform (google forms) but were completed on paper by the clinical group, either at the healthcare units, in the case of inpatients, or at home, in the case of outpatients. The socio-demographic information for both groups is shown in Table 1, the linguistic profile in Fig 1, and mean scores obtained on the screeners in Table 2. Within the clinical group, a total of 30 individuals (29.41%) had a main diagnosis of a psychotic disorder (F20) according to the ICD-10, 17 (16.67%) of an affective disorder (F30), 16 (15.69%) of phobias or adaptive disorders (F40), 13 (12.75%) of substance use disorders (F10), 5 (4.90%) of eating or sleep disorders (F50) and 1 (0.98%) of a developmental disorder (F80).

**Table 1:** Description of the sample.

	General group N = 521 N (%)	Clinical group N = 102 N (%)
Age [mean (sd)]	26.53 (7.74)	30.65 (8.32)
Sex		
Male	131 (25.14)	51 (50)
Female	383 (73.51)	50 (49.02)
Others	7 (1.34)	1 (0.98)
Marital status		
Single	359 (68.91)	79 (77.45)
Married/living together	106 (20.35)	14 (13.73)
Separated/divorced	3 (0.19)	7 (6.86)
Widowed	1 (0.19)	2 (1.96)
Others	52 (9.98)	0 (0)
Level of education		
None	0 (0)	1 (0.99)
Primary	2 (0.38)	1 (0.99)
Secondary	2 (0.38)	26 (25.74)
High School	96 (18.43)	12 (11.88)
Professional	37 (7.10)	37 (36.63)
University Degree	384 (73.70)	24 (23.76)
Occupation		
Employed/studying	436 (83.69)	55 (54.46)
Unemployed	40 (7.68)	38 (37.62)
Housekeeper	11 (2.11)	0 (0)
Pensioner	1 (0.19)	8 (7.92)
Others	33 (6.33)	0 (0)

sd: Standard deviation.

**Table 2:** Symptom severity across questionnaires and groups.

PQB total	General group	Clinical group	General vs. Clinical group (matched groups)	
Mean (sd)	3.1 (3.4)	5.4 (4.1)	2.9 (3.2)	5.4 (4.1)
Min-max	0–16	0–17	0–13	0–17
**PQB distress**				
Mean (sd)	9.2 (11.7)	17.5 (15.7)	7.8 (10.4)	17.5 (15.7)
Min—max	0–58	0–65	0–43	0–65
**PHQ-9**				
Mean (sd)	7 (5.1)	14.8 (7.9)	6.3 (5.1)	14.8 (7.9)
Min-max	0–29	1–30	0–26	1–30
**GAD-7**				
Mean (sd)	6.4 (3.9)	10.3 (5.1)	5.9 (3.6)	10.3 (5.1)
Min-max	0–21	0–21	0–21	0–21
**DASS total**				
Mean (sd)	18.6 (19)	58.2 (31)	18.3 (19.6)	58.2 (31)
Min-max	0–105	2–120	0–88	2–120
**DASS depression**				
Mean (sd)	6.2 (8.3)	21.6 (13.3)	5.6 (8.2)	21.6 (13.3)
Min-max	0–42	0–42	0–42	0–42
**DASS anxiety**				
Mean (sd)	4.5 (5.6)	16.4 (10.4)	4.6 (5.2)	16.4 (10.4)
Min-max	0–38	0–42	0–25	0–42
**DASS stress**				
Mean (sd)	7.9 (7.2)	20.3 (10.7)	8.1 (7.5)	20.3 (10.7)
Min-max	0–38	0–42	0–31	0–42

PQB: Prodromal questionnaire-Brief; PHQ-9: Patient Health questionnaire-9; GAD-7: General Anxiety Disorder Scale-7; DASS: Depression, Anxiety and Stress Scale; sd: standard deviation; min: minimum score; max: maximal score.

Participation was restricted to individuals between 18 and 45 years of age, to ensure that they were familiar with standard Basque, which was used in the translation of the instrument. There are several dialects of Basque, which differ considerably in both grammar and vocabulary forms, and the diffusion of standard Basque was not generalized until 1975.

Participants were dropped from the analysis of the general and clinical group data sets in the following cases:

- unlikely socio-demographic information entered (e.g., age indicated as 3 years, 1 participant).
- Incomplete information about language profile (7 participants in the general population; 6 in the clinical population).
- Failure to complete a questionnaire (variable, see results for details).
- Basque or Spanish indicated as L3 (1 participant).

Data were collected between November 1, 2019 and January 31, 2021. Participants in both groups were informed about the ongoing research and gave their written informed consent before participating. All procedures were approved by (CEIm-E) Ethics Committee [PI2019052].

### 2.2. Instruments

#### BCBL language competence questionnaire

This is a questionnaire developed by the BCBL to make a comprehensive linguistic assessment of an individual. It collects information on: the languages that a person speaks, in order of preference; the percentage of time they are exposed to each language they know (i.e., percentage of time they read, listen to, write and speak in each known language, total time being 100%); the age of acquisition of each language; self-estimated proficiency in each language in different areas (speaking, listening, reading, writing) on a scale of 0 to 10; whether they have official qualifications in any of the known languages; as well as data on handedness, the first language of the parents, and the length of any experience living abroad. A copy of the questionnaire can be found in the Supplementary Materials (SM).

#### Prodromal Questionnaire-Brief Version(PQ-B) [38]

This is a self-report psychosis risk screening measure with 21 items and a yes/no dichotomous response structure (frequency scale). PQ-B was developed from the original 92-item Prodromal Questionnaire [39]. Following each individual item, and when the response is affirmative, a follow-up question inquiring about frequency and related distress/impairment is included, rated on a 5-point Likert scale (distress scale). Different cut-off points in the frequency and distress scales have been proposed in the different populations in which the instrument has been validated [27–29, among others]. The validations conducted in the Spanish population propose a cut-off score of >9 in the frequency scale [40] and >29 for the distress scale [41]. These validation studies have shown that the instrument has a good internal consistency, with alpha values ranging between 0.83 and 0.89, and a unidimensional factorial structure.

#### Patient Health Questionnaire (PHQ-9) [33]

This is a 9-item self-report measure with a response option rated on a 4-point Likert scale that explores the nine DSM-IV diagnostic criteria for depressive disorders. There is a single final item that evaluates functional impairment. The cut-off point of ≥10 has been established as indicating a possible depressive disorder. It has been validated in several languages and cultural contexts [e.g., 41–44], with good internal consistency, as indicated by alpha values between 0.79 and 0.89.

#### Generalized Anxiety Disorder Scale (GAD-7) [36]

This is a self-report measure for evaluating the presence and severity of GAD during the past two weeks, according to the DSM-IV-TR criteria. It consists of 7 items with a response option rated on a 4-point Likert scale. A score ≥10 has been proposed as the cut-off point for identifying cases of GAD. Cut-off points of 5, 10, and 15 might be interpreted as representing mild, moderate, and severe levels of anxiety. It has been validated in different languages and populations [e.g., 45–47], reporting an excellent internal consistency, with alpha values between 0.85 and 0.95.

#### Depression, Anxiety and Stress Scale-42 (DASS-42) [37]

This is a self-administered questionnaire composed of 42 items, which measures severity of depression, anxiety and stress over the last week, using a 3-point Likert scale. Cut-off scores of 10 for depression, 8 for anxiety and 15 for stress have been proposed. It has been validated in many different languages [e.g., 48–51], with reported alpha values between 0.84 and 0.97. The three-factor solution, corresponding to the depression, anxiety, and stress scales, has been supported in most studies.

### 2.3. Validation procedures

The Basque versions of the questionnaires were developed through the translation and back-translation method. Back-translation methodology ensures the most accurate translation and an improved reliability and validity of the process [52]. Translation from English to Basque was conducted by the Translation Service of Osakidetza-Basque Health Service (V1). The first translated Basque version was revised by a trilingual English-Spanish-Basque psychiatrist to ensure that technical terms were correct (V2). The revised version was then back-translated into English by an external professional translation service (V3). V3 were sent to the authors and, after their approval, V2 was revised again by a Basque technician, in order to ensure comprehensibility and feasibility of administration for the general population (V4). V4 was considered the final version in all cases. All necessary permissions were obtained to translate and publish the questionnaire.

### 2.4. Data analysis

Firstly, the psychometric properties of the questionnaires were analyzed, including sensitivity, confirmatory analysis, reliability, concurrent validity, and factor loadings.

Secondly, we analyzed the association between participants’ linguistic profile and symptomatic expression, by means of regression models in which scores on the four questionnaires were used as dependent variables and predictor variables were four linguistic profile measures: AoA of Basque, Exposure to Basque, Proficiency in Basque and L1. Demographic variables (age, gender, education) were also included in the model as predictors. The distributions of the linguistic profile in the general and in the clinical group are illustrated in Fig 1.

The Education predictor originally included 6 categories: none, primary, secondary, high school, professional and university degree, with a non-homogeneous stratification of participants across the 6 categories. Since this was problematic for the computation of coefficients in interactions terms (i.e., full regression models returned NAs and/or the algorithm did not converge), educational categories with less than 10 participants were dropped from the analysis. Specifically, the categories “primary” and “secondary” education were dropped from the analysis of the general group (2 participants in each category), while “none” and “primary” (1 participant each) were removed from the Education predictor in the analysis of the clinical group. Similarly, “other” for the Gender variable was dropped from the analysis of both the general and the clinical group (7 and 1 participants, respectively). In reporting each analysis, the number, age and gender of the participants in each group is provided.

Multiple-regression models with Poisson distribution were fitted for each dependent variable using R version 4.2.1 [53]. To reduce overdispersion of the data (as checked by the check_dispersion function in the *Performance* package) [54], negative binomial regression models were applied and their likelihood compared to a simple Poisson regression model (package pcsl) [55]. Overdispersion tests showed that all negative binomial models had a Chi-Square test statistic greater than the critical 2.7055 value at alpha = .05 (all *p*s < .001), indicating that data were more likely under a negative binomial than a simple Poisson regression model, so negative binomial regression models are reported below.

For each dependent variable, the effect of each demographic and linguistic profile variable was tested in three different sets of analysis: within the general group, within the clinical group and between groups. For the between-group analysis we subset a sample of Gender- and age-matched participants from the two groups. Since the two groups could not be matched in Education, this variable was included in the analysis as a covariate. Patient-general group matching was carried out with the *match* function from the R package MatchIt [56].

For both within- and between-group analyses, maximal models were built in which all four linguistic and three demographic variables, as well as their interaction, were simultaneously entered. The R model syntax for the two within-group analyses is indicated below in 1, while 2 shows the maximal model adopted for the between-group analysis. Full model structure was simplified whenever non-convergence problems arose, with such changes noted below in the corresponding results section. The complete output of each maximal regression model fitted is reported in Supplementary Tables 4–7 in S1 File.

1. glm.nb (y ~ AoA + Proficiency + Exposure + L1+ Age + Gender + Education +
     AoA*(Age + Gender + Education) +
     Proficiency * (Age + Gender + Education) +
     Exposure * (Age + Gender + Education) +
     L1 * (Age + Gender + Education), data,
     general = glm.control(maxit = 250))
2. glm.nb (y~ Group +AoA + Proficiency + Exposure + L1 + Education +
     Group * AoA+
     Group * Proficiency+
     Group * Exposure+
     Group * L1, data, control = glm.control(maxit = 250))

To avoid collinearity among predictors, continuous variables (Age, AoA, Exposure to Basque and Proficiency in Basque) were converted to z-scores. The vif function (R package CAR) was used to check whether the GVIF^^(1/(2*Df)^ values of predictors and their interactions exceeded 10 (all values < 5).

A backward model selection procedure (R package MASS) [57] was used to select the combination of explanatory variables included in the maximal model that provided the lowest AIC. The overall effect of each variable was determined through an Analysis of Deviance using the R function Anova (Type III, Wald’s test, R package “car”) [58]. Due to the significant positive correlation among the scores obtained from the 4 screeners, for each dependent variable, p-values obtained from the between-group and the two within-group analyses were FDR corrected (separately for the three groups of analysis). We report the effect of predictor variables and interactions that survived FDR correction below, while the reader is referred to the Supplementary Materials for the complete output of stepwise regression models (Supplementary Tables 4–7 in S1 File).

Estimated marginal means (EMM, henceforth) were calculated for comparisons between the general and the clinical group as well as for comparisons between levels of significant categorical predictors (L1, Gender, Education) using the R package *emmeans* [59]. Results of post-hoc contrasts were FDR-adjusted for multiple comparisons. For each contrast, we report EMM, standard error (SE), confidence intervals (CI), *z* ratios and adjusted p-values. To increase the interpretability of results, EMM and figures are reported using untransformed values of Age, AoA, Proficiency and Exposure.

## 3. Results

Table 2 provides the scores obtained on the different psychometric scales for the general and clinical groups.

Of the 512 participants in the general group and 96 participants in the clinical group, a variable number of participants failed to complete all four questionnaires (PQB: 26 in the clinical group; PHQ-9: 2 in the general group; 3 in the clinical group; DASS: 8 in the clinical group).

### 3.1. Psychometric properties

The Basque versions of the scales showed adequate sensitivity to discriminate general and clinical groups, with p-values < .0001 for both mean and median comparisons. Confirmatory analysis yielded adequate goodness-of-fit indices (Supplementary Table 1 in S1 File). The cut-off for comparative fit index (CFI) was set at > 0.95 and for Tucker-Lewis index (TLI) t > 0.95 [60]. The cut-off for acceptable model-fit was set at RMSEA < 0.08 [61]. Reliability and concurrent validity data are shown in Supplementary Table 2 in S1 File, with Cronbach’s alpha values ranging from .71 and .75 for PHQ-9 factor 2 to .90 for the stress subscale of the DASS-42. All alpha values were above 0.7, demonstrating good reliability properties. Factor loadings for all the scales are represented in Supplementary Tables 3A-D in S1 File. In this case, previous factor loadings were confirmed for DASS, PQB and GAD-7; in the case of PHQ-9, a 2-factor structure fitted better than the unifactorial model.

### 3.2. Differences in symptom severity according to linguistic variables

Below, we report the results of the three analyses (within-group analyses of the general and clinical populations and between-group analysis) conducted to assess the relation between reported symptom severity on the four questionnaires and four linguistic predictor variables (L1, Basque proficiency, exposure and AoA). For the PQB, we report the models with the total scores and with the distress ratings and for the DASS, we report the total score and the three elements separately (depression, anxiety and stress).

#### PQB total

*General population*. The analysis of the general group included 501 participants (373 female; mean age: 26.6, standard deviation (SD: 7.2, range: 18–45). The model with lowest AIC included Exposure, Proficiency and L1 among the linguistic predictors, and the three demographic predictors, along with the interaction between Exposure and Gender and between L1 and Education. Among these, only Education (Df_(2),_ Chi-Square: 13.761, corrected p = 0.02190) and Age (Df_(1),_ Chi-Square: 8.841, corrected p = 0.02660) were significantly associated with PQB total scores, while the effect of linguistic variables did not reach statistical significance.

*Clinical population*. The clinical group included 70 participants (34 female; mean age: 30.4 years, SD: 8.2, range: 18–45). The model with the lowest AIC included Exposure, Age and the interaction between these two predictors. Neither Exposure nor interaction between Exposure and Age reached statistical significance.

**Fig 2:**
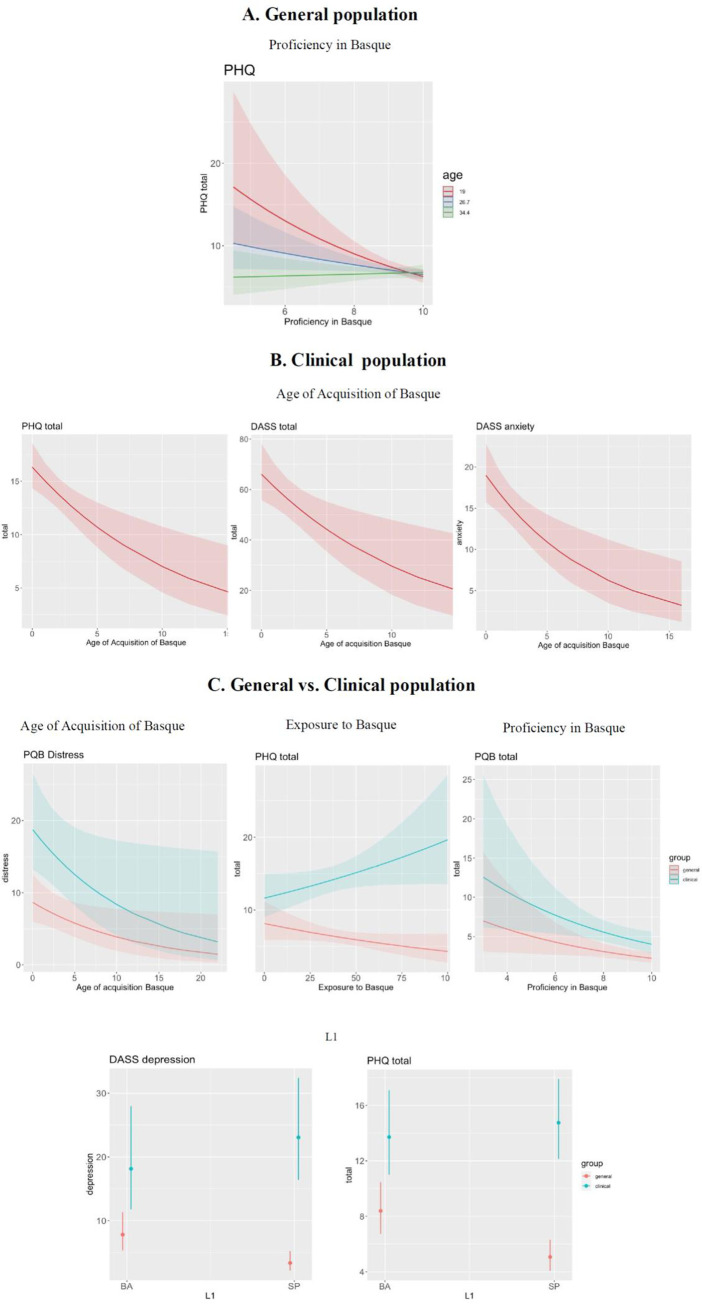
Association between expressive symptoms and linguistic variables. (A) General population, (B) Clinical population (C), Comparison between General and Clinical populations. Note that in Fig 2A, age is a continuous predictor that has been broken down into three age groups only for illustrative purposes. PHQ: Patient Health Questionnaire; DASS: Depression, Anxiety and Stress Scale; PQB: Prodromal questionnaire.

*Between-group analysis*. A total of 72 age- and gender-matched participants per group were included in this analysis (General group: 34 females; mean age: 30.35 years, SD: 7.97; Clinical group: 34 females; mean age: 30.41; years, SD: 7.96). The model with the lowest AIC included Group, AoA and Exposure. Group was significantly associated with PQB symptoms, as expected (Df_(1)_, Chi-square: 13.965; corrected p = 0.0008). Significantly higher total PQB scores were found in the clinical group (EMM: 5.02, SE: 0.609, CI: 3.96, 6.36) compared to the general population (EMM: 2.95, SE: 0.382, CI: 2.29, 3.81; z-ratio: -2.930, p = 0.0034). Proficiency in Basque was also associated with PQB scores (Df_(1)_, Chi-Square: 7.536, corrected p = 0.0181): for each 1 point increase in Basque Proficiency (on a scale of 0 to 10), PQB scores decreased by -0.17 (intercept: 2.63, SE: 0.06444 CI: -0.30, 0.03) (see Fig 2).

Stepwise regression analysis for the PQB scale (total score) is shown in Supplementary Table 4A in S1 File.

#### PQB distress

*General population*. The analysis of the general group included a total of 501 participants (373 females; mean age: 26.65, SD: 7.73, range: 18–45). A model including Education and Age provided the lowest AIC. Education was found to be a significant predictor of PQB scores (Df_(2)_ Chi-Square: 14.400; corrected p = 0.02190).

*Clinical population*. The analysis of the clinical group included a sample of 68 participants (33 females; mean age: 30.28 years, SD: 8.03, range: 18–45 years). The intercept-only model had the lowest AIC.

*Between-group analysis*. A total of 68 age- and gender-matched participants per group were included in this analysis (General group: 34 females; mean age: 30.2 years, SD: 8; Clinical group: 34 females; mean age: 30.3; years, SD: 8). A model including Group, AoA, Proficiency and Exposure provided the lowest AIC. Group, AoA and Proficiency were found to be significantly associated with distress symptoms. Group was significantly associated with distress symptoms (Df_(1)_, Chi-Square: 14.498, corrected p = .0007), with lower scores in the general group. A significant association was found between AoA and distress symptoms (Df_(1)_, Chi-Square: 5.547, corrected p = .0407). For each year later the AoA of Basque, distress symptoms across the two groups decreased by 0.24 points (SE: 2.01; CI: -0.45, -0.01), as shown in Fig 2. Similarly, Proficiency was associated with distress scores (Df_(1)_, Chi-Square: 8.601, corrected p = 0.01110). For each 1 point increase in Basque Proficiency, participants’ distress symptoms across the two groups decreased by -0.47 points (SE: 0.16, CI: -0.82, -0.14) (Fig 2C).

Stepwise regression analysis for the PBQ distress subscale is shown in Supplementary Table 4B in S1 File.

#### PHQ-9

*General population*. The analysis of the general group included a final sample of 499 participants (371 females; mean age: 26.69, SD: 7.28, range: 18–45). The model providing the lowest AIC included the predictors Exposure, Proficiency, L1, Education, and Age, as well as the interaction between Exposure and Age, L1 and Education, and Proficiency and Age. After FDR correction, only the interaction between Proficiency and Age (Df_(1)_, Chi-Square: 8.838, corrected p = 0.02660) was found to be reliably associated with PHQ-9 scores. As shown in Fig 2, participants below 26 years and with lower proficiency in Basque reported more severe depressive symptoms compared to older participants.

*Clinical population*. Ninety-one participants were included in the analysis of the clinical group (47 females; mean age: 30.87 years, SD: 8.08, range: 18–45 years). Stepwise regression indicated that the lowest AIC was provided by a model including AoA, Exposure, Proficiency, L1, Age, Gender, Education, as well as the interaction between Proficiency and Gender, L1 and Gender, AoA and Age, Exposure and Age, and Exposure and Education. After FDR correction, only AoA was found to be a significant predictor (Df_(1)_, Chi-square: 11.552, p = 0.00940). For each year later the AoA of Basque, participants’ PHQ scores decreased by -0.23 (intercept: 2.78, se: 0.15, CI: -0.38, -0.08 (Fig 2).

*Between-group analysis*. A total of 91 age- and gender-matched participants per group were included in this analysis (General group: 51 females; mean age: 31.56 years, SD: 8.39, range: 18–45; Clinical group: 51 females; mean age: 30.87; years, SD: 8.08, range: 18–45). The lowest AIC was provided by a model that included Group, AoA, Proficiency and L1, together with the interaction between Group and AoA, Exposure and L1.

L1 was significantly associated with PHQ scores (Df_(1)_, Chi-Square: 9.120, corrected p = 0.00930). Participants whose L1 was Basque had higher PHQ scores (EMM: 10.98, se: 0.813, CI: 9.50–12.69, Fig 2) compared to those with Spanish as their L1 (EMM: 8.63, se: 0.589, CI: 7.55–9.86, z-ratio: 2.268, p = 0.0234). Moreover, a significant L1 by Group interaction was found (Df_(1)_, Chi-Square: 6.305, corrected p = 0.03310). Post-hoc analysis revealed that within the clinical group, having Basque or Spanish as L1 was associated with symptoms of similar severity (Basque: 13.60, se: 1.639, CI: 10.74–17.22; Spanish: 14.67, se: 1.291, CI: 12.35–17.43, z-ratio: -0.461 p = 0.6449), while in the general group, participants with Basque as their L1 reported higher symptoms compared to those with Spanish as their L1 (Basque: 7.83, se: 0.797, CI: 6.42–9.56; Spanish: 4.67, se: 0.554, CI: 3.70–5.90, z-ratio: 3.020, p = 0.0025). A significant Group by Exposure interaction was also found (Df_(1)_, Chi-Square: 5.915, corrected p = .03810). Post-hoc analyses revealed a marginal effect of Exposure to Basque within the Clinical group, such that for each 1% increase in exposure to Basque, symptoms increased by 0.13 points (intercept: 2.82, se: 0.08, CI: -0.017–0.28, z-value: 1.70, p = 0.0887). In contrast, in the general group, symptoms decreased by 0.14 points (intercept: 2.07, se: 0.09, CI: -0.33, 0.04, z-value: -1.60, p = 0.1076) for each 1% increase in exposure to Basque (Fig 2).

Stepwise regression analysis for the PHQ-9 is shown in Supplementary Table 5 in S1 File.

#### GAD-7

*General population*. A final sample of 501 participants was included in the analysis of the general group (373 females; mean age: 26.66 years, SD: 7.28, range: 18–45). The model with lowest AIC included the following predictors: AoA, Exposure, Proficiency, Age, Gender, and the interactions between Age and AoA, Proficiency and Exposure. However, none of these predictors were found to be significantly associated with GAD symptoms after FDR correction.

*Clinical population*. The clinical group included 93 participants (47 females; mean age: 30.62, SD: 8.16, range: 18–45). The model with the lowest AIC included only the Gender variable. However, Gender was not significantly associated with GAD-7 scores.

*Between-groups analysis*. A total of 93 age- and gender-matched participants per group were included in this analysis (General group: 51 females; mean age: 31.4 years, SD: 8.6, range: 18–45; Clinical group: 47 females; mean age: 30.6; years, SD: 8.2, range: 18–45). A model including only the Group predictor provided the lowest AIC. Group was significantly associated with GAD symptoms (Df_(1)_, Chi-Square = 46.882, corrected p < .001) Participants in the general group (EMM: 5.87, SE: 0.361, CI: 5.20–6.62) reported lower anxiety symptoms, as expected.

Stepwise regression analysis for the GAD-7 is shown in Supplementary Table 6 in S1 File.

#### DASS

*DASS total*. *General population*. A total of 509 participants were included in the analysis of the general group (373 females; mean age: 26.6, SD: 7.3, range; 18–45). The maximal model tested included all the linguistic and demographic predictors, as well as the interactions between all four linguistic predictors and Gender and Age. The interaction with Education was removed since it led to non-convergence of the algorithm. The lowest AIC was provided by a model including the following predictors: Age and Education; Proficiency and L1; and the interaction between Proficiency and Age. However, none of these predictors were significantly associated with DASS total scores after FDR correction.

*Clinical population*. A total of 89 participants were included in the analysis of the clinical group (41 females; mean age: 30.4 years, SD: 7.9, range: 18–45). The model with lowest AIC included AoA, L1, Age and Education, as well as the interaction between AoA and Age. After FDR correction, AoA was found to be a significant predictor of total DASS scores (Df_(1)_, Chi-Square = 7.970, corrected p = 0.03330). One year later AoA of Basque was associated with a decrease in DASS total scores of 0.22 points (intercept: 3.76, SE: 0.08, CI: -0.4, -0.05, Fig 2).

*Between-group analysis*. A total of 86 age- and gender-matched participants per group were included in this analysis (General group: 41 females; mean age: 30.3 years, SD: 8, range: 18–45; Clinical group: 41 females; mean age: 30.4 years, SE: 8, range: 18–45). Backward model selection indicated that the lowest AIC was provided by a model including Group, AoA and L1, and the interactions between Group and AoA and L1. Group was significantly associated with total DASS scores (Df_(1)_, Chi-Square: 23.3869, corrected p < .001), as expected. No other significant association was found.

Stepwise regression analysis for the DASS-42 (total score) is shown in Supplementary Table 7A in S1 File.

#### DASS depression

*General population*. A total of 501 participants were included in the general group (373 females; mean age: 26.7, SD: 7.3, range: 18–45). In the full model, inclusion of the interaction between linguistic predictors and Education resulted in non-convergence of the algorithm and so this term was dropped. Backward model selection indicated that a model with Exposure, Proficiency, L1, Education, Age and the interaction between Proficiency and Age provided the lowest AIC. A significant association was found between Education and depression scores (Df_(2)_ Chi-Square: 13.059, corrected p = 0.02190), while the effects of linguistic variables did not reach statistical significance.

*Clinical population*. A total of 87 participants were included in the analysis of the clinical group (42 females; mean age: 30.38 years, SD:7.95, range: 18–45). The intercept-only model provided the lowest AIC.

*Between-group analysis*. A total of 87 age- and gender-matched participants per group were included in this analysis (General group: 42 females; mean age: 30.3 years, SD: 8.2, range: 18–45; Clinical group: 42 females; mean age: 30.4 years, SD: 13.3, range: 18–45). Stepwise regression showed that a model with Group, AoA, Proficiency, Exposure, L1 and the interaction between Group and AoA and Exposure and L1 provided the lowest AIC. Group was significantly associated with depression symptoms (Df_(1)_, Chi-Square: 67.947, p < .001), with lower scores for the general group (EMM: 5.63 SE: 0.668; CI: 4.46,7.11). Participants’ L1 was also associated with depression symptoms (Df_(1)_, Chi-Square: 5.754, corrected p = 0388). Moreover, a significant interaction between Group and L1 (Df_(1)_, Chi-Square: 5.136, corrected p = .0483) showed that within the clinical group, the severity of depression symptoms was similar among participants with Basque (EMM: 18.69, SE: 3.784, CI: 12.57, 27.79) and Spanish as L1 (EMM: 22.94, SE: 3.547, CI: 16.94, 31.06, z-ratio: - 0.731, p = 0.4647). In contrast, within the general group, participants whose L1 was Basque reported higher depressive symptoms (EMM: 7.24, SE: 1.266, CI: 5.13, 10.20) compared to those with Spanish as their L1 (EMM: 3.54, SE: 0.723, CI: 2.37–5.28, z-ratio: 2.399, p = 0.0165, see Fig 2)

Stepwise regression analysis for the depression subscale of DASS-42 is shown in Supplementary Table 7B in S1 File.

#### DASS anxiety

*General population*. A total of 501 participants were entered in the analysis for the general group (373 females; mean age: 26.66, SD: 7.73, range: 18–45). Backward model selection indicated that a model including Education and Age provided the lowest AIC. A significant association was found between Education and anxiety scores (Df_(2)_, Chi-Square: 10.296, corrected p = 0.04360).

*Clinical population*. A total of 86 participants were included in the analysis of the clinical group (41 females; mean age: 30.4 years, SD: 8, range: 18–45). The model with the lowest AIC resulting from backward selection included AoA, Exposure, Education, Age and the interaction of Age with AoA and Exposure. As shown in Fig 2, AoA correlated with anxiety symptoms in the clinical group (Df_(1)_, Chi-Square: 10.819, corrected p = 0.00940: as participants’ age increased, anxiety symptoms decreased by 0.32 points (intercept: 3.05, se: 0.14643, CI: -0.51, -0.10).

*Between-group analysis*. A total of 86 age- and gender-matched participants per group were included in this analysis (General group: 41 females; mean age: 30.3 years, SD: 8, range: 18–45; Clinical group: 41 females; mean age: 30.4 years, SD: 8, range: 18–45). The model with the lowest AIC included the Group variable. Group was significantly associated with anxiety scores (Df_(1)_, Chi-Square: 82.043, corrected p < .001), with the general group showing lower anxiety (EMM: 4.63, SE: 0.477; CI: 3.78,5.66) than the clinical group (EMM: 16.38, SE: 1.540; CI: 13.63, 19.70).

Stepwise regression analysis for the anxiety subscale of DASS-42 is shown in Supplementary Table 7C in S1 File.

#### DASS stress

*General population*. A total of 501 participants were included in the analysis of the general group (373 females; mean age 26.66, SD: 7.73, range: 18–45). Backward model selection resulted in a model that included Proficiency, L1, Education, Age, the interaction between Age and Proficiency and between L1 and Education. None of the variables showed a statistically significant association with stress symptoms.

*Clinical population*. The final sample for the analysis of the clinical group included 87 participants (42 females; mean age: 30.55, SD: 8.1, range: 18–45). The lowest AIC was provided by the intercept-only model.

*Between-groups analysis*. A total of 87 age- and gender-matched participants per group were included in this analysis (General group: 42 females; mean age: 30.5 years, SD: 8.1, range: 18–45; Clinical group: 42 females; mean age: 30.6 years, SD: 8.1, range: 18–45). A model with the Group predictor provided the lowest AIC, with the variable Group being significantly associated with stress symptoms (Df_(1)_, Chi-Square: 57.768, corrected p < .001). Higher stress symptoms were found in the clinical population (EMM: 20.28, SE: 1.684, CI: 17.2, 23.9) compared to the general population group (EMM: 8.08, SE: 0.712, CI: 6.8, 9.6; z-ratio: -7.600, p < .001)

Stepwise regression analysis for the stress subscale of DASS-42 is shown in Supplementary Table 7D in S1 File.

## 4. Discussion

The goal of the current study was to develop and test the psychometric properties of the Basque versions of the PQ-B, the PHQ-9, the GAD-7 and the DASS-42, as well as to assess whether and to what extent the severity of psychopathology could be associated with linguistic profile, in a representative sample of Basque-Spanish bilingual individuals.

Following back-translation, the validation of the four questionnaires revealed their adequate sensitivity and goodness of fit, as well as good reliability indices.

Based on previous studies [6–12], we expected participants with Basque as their L1 to report more severe symptoms compared to those whose L1 was Spanish, both in the general and in the clinical population, as well as in the comparison between them. A similar pattern was expected for participants with greater exposure to Basque and proficiency in this language, and those who had acquired Basque earlier in life. However, the results reveal a non-homogeneous scenario across populations and questionnaires: whereas reporting of depressive symptoms appeared to be modulated by all four linguistic variables analyzed with a congruent pattern, the severity of psychotic symptoms was associated only with proficiency and age of acquisition of Basque, and furthermore, these associations contradict previous findings. In both cases, a relation between linguistic variables and reported symptoms consistently emerged in between-group analysis, while it was less reliable in the two within-group analyses. Below, we discuss these results and their implications in greater detail.

### 4.1. Differences in prodromal psychotic symptom severity in relation to linguistic variables

Contrary to what we expected, linguistic variables were not consistently associated with the psychotic symptomatology evaluated by the PQB, either in the general or the clinical group separately. Although the existing literature is scarce, most previous reports exploring the differences in psychotic symptoms based on the language of assessment, including a meta-analysis conducted by our group [5], pointed to reporting of more severe symptoms when the assessment was conducted in the L1 compared to L2. In the current study, L1 was not significantly associated with total or distress scores on the PQB. Indeed, the only linguistic variable associated with the PQB total score was proficiency, and the direction of the association (higher proficiency, lower score) seem to contradict what previous studies have described. Also, none of the other linguistic variables that could be linked to higher proficiency (AoA, exposure and L1) seem to be related to the PQB total score, which is the score that best reflects the presence of psychotic symptomatology.

A plausible explanation for these contrasting results concerns the different socio-linguistic contexts in which this and previous studies were conducted. Participants in both our general and clinical samples were highly proficient bilingual speakers (see Fig 1) who, in most cases, acquired both languages early in their lives and, at the time of testing, lived in a balanced bilingual context. In contrast, most previous studies were conducted in monolingual settings with a large migrant population, whose first language was different from the language in which the evaluation was carried out [6,8,10,20].

In addition, most investigations have not separately considered linguistic factors such as proficiency, AoA, or exposure to each language. In this respect, our work is more rigorous since it included these different linguistic factors and, thus, it could be considered a more complete examination of the linguistic reality of the participants.

The comparison of the two groups revealed significant associations between Basque Proficiency and AoA with the symptoms evaluated by the PQB questionnaire. In both groups, the higher participants’ proficiency in Basque, the lower their psychotic symptoms, as evaluated by the PQB total. In contrast, the later the age at which Basque was acquired, the lower the distress symptoms reported by participants in the two groups. Proficiency is usually higher in the language mainly used at home [62], so the association between a greater proficiency in Basque and less severe symptoms was unexpected. It seems at odds with the association between the later age of Basque acquisition and lower distress symptoms observed in the same samples. Therefore, these results require further study.

### 4.2. Differences in depressive and anxious symptom severity according to linguistic variables

Regarding depressive and anxious symptomatology (measured with PHQ-9, GAD-7 and DASS), our result point to a greater influence of linguistic variables on depressive symptoms than on anxious ones. Indeed, with respect to anxious symptomatology, the effect of linguistic variables is marginal and not consistent across the different tools used to assess it.

Specifically, and although we failed to find evidence for the association between participants’ L1 and their depressive symptoms when the two groups were analyzed separately, in the between-group analyses, the L1 was differentially associated with the severity of the depressive symptoms reported by the two groups. In both PHQ-9 and DASS depression, L1-Basque participants in the general group reported more severe depressive symptoms compared to those with Spanish as their L1, as we expected. In contrast, in the clinical group, the difference in symptom severity between L1-Basque and L1-Spanish participants was not statistically significant. In other words, whether the assessment was carried out in the participants’ first language mattered only in the non-clinical population. This difference between both groups may be due to the fact that the instruments used have screening purposes, so that there could be a ceiling effect in the results obtained in the clinical group that prevents adequate discrimination. Although the individuals included in the clinical sample have different underlying pathologies, it is to be expected that they report greater psychological distress as a group. Nonetheless, a later AoA of Basque was associated with lower scores on PHQ-9 in the clinical group, which could be in line with the above-mentioned results.

Finally, the amount of exposure to Basque was associated with different PHQ-9 symptoms across the two groups. While in the clinical group greater exposure to Basque was associated with increased depressive symptoms, this was not the case in the general group. This result seems to contradict the previous one, although it is true that we cannot infer that having Basque as L1 is equivalent to a greater use in everyday life. Use and exposure depend to a large extent on the specific context in which an individual lives and works/studies. Exposure was a composite measure that included different components (the percentage of reading, writing, speaking, listening in a specific language), so it could be interesting to make a disaggregated analysis of the relationship with each of these components in the future. It is possible that the associations are different depending on the area of language use. It should also be noted that we assessed symptomatology by means of written questionnaires, which involve reading and writing, and their results may not generalize to interview-based assessment, which involves speaking and listening, and thus a more spontaneous and less formal type of communication. Furthermore, the involvement of the three main executive functions [63] in oral communication and written communication differ [64]. In relation to verbal communication, a multidirectional relationship between language and executive functions has been described [65], so the impact of L1 on these different skills may differ.

There are no precedents in the literature on differential reporting of depressive and anxiety symptoms according to different linguistic variables. Based on our results, we could state that first language is a clinically relevant variable at the time of assessing affective symptoms in the general population, in line with previous investigations into the assessment of psychotic symptoms.

### 4.3. Limitations and strengths

The main strengths of the study are its sample size, its representativeness (including general and clinical populations) and its detailed analysis of linguistic profile variables, which has not been carried out before. In addition, to our knowledge, this is the first study that has explored possible differences in symptomatic severity in relation to a variety of linguistic factors. Crucially, in contrast to previous studies, which have focused solely on psychotic symptoms, we also examined the role of language in depressive and anxious symptomatology. Moreover, to our knowledge, this is also the first time that differences in symptomatic reporting in relation to L1 have been studied by means of written measures (psychometric tools) instead of interviews.

However, one limitation of this study is precisely the use of written assessment tools, which prevents us from directly comparing our results to existing studies conducted through clinical and semi-structured interviews. In this study, we exploited the need to develop and validate Basque language versions of various screeners to simultaneously test differences in the reporting of psychopathology as a function of linguistic variables in bilingual populations. However, it would be interesting to design a new study to explore possible differences between written instruments and interviews, in a balanced bilingual population, using both languages.

In addition, it should be highlighted that we tested a balanced bilingual population, with most Basque speakers living in the Basque Country having high-proficient bilingual status. Spanish is the language used most extensively outside of the family environment and by mainstream press and television, so even those individuals whose first language is Basque achieve a high level of proficiency in Spanish by adulthood. This situation is similar to other settings in which two native languages coexist but one of them is dominant, such as the French-speaking area of Canada or Wales (in the United Kingdom). Therefore, our results may be generalizable to these types of linguistic settings, but might differ from those obtained with migrant populations whose first language is different from the language of assessment.

Finally, the fact that we found no language-related differences in the self-reports of psychotic symptomatology in high proficient bilinguals does not indicate that, even in high proficient bilinguals, there may be no differences in the verbal expression of psychotic symptoms when interviewing in L1 vs. L2. This would be an important line for future research.

This study is a first approach to a very relevant topic, if we take into account that most of the world’s population is bi- or multilingual, and that there are many societies with various official languages, where linguistic choice in healthcare is viewed as an important topic.

### 4.4. Clinical implications

In contrast to our expectations based on our previous meta-analysis [5], in the present study we did not find differences in the severity of reported prodromal psychotic symptomatology according to the language of assessment. Specifically, our results suggest that in highly proficient bilingual populations, whether assessments of psychotic symptoms by means of self-reported questionnaires are conducted in the first *vs*. second language may not be of clinical relevance. This has important clinical implications since it suggests that availability of bilingual psychometric tools to assess psychotic symptomatology may not be critical for treatment outcomes in balanced bilingual sociocultural contexts, even if healthcare availability in the co-official language chosen by the patient should be viewed as a linguistic right.

However, for the questionnaires assessing depressive symptoms, although the L1 was not a reliable predictor in within-group analyses, it was found to differentiate the clinical group from a matched group of controls when their depressive symptomatology was contrasted directly, along with independent effects of proficiency, exposure and age of language acquisition. In particular, an association of L1-Basque in the between-group analyses of both PHQ-9 and DASS-depression, and of AoA in the PHQ-9 clinical group, with reported severity were found for depressive symptomatology.

Thus, and in contrast to prodromal psychotic symptomatology, the language in which the self-reported assessment of affective symptomatology was carried out had relevant clinical implications, mainly in the general population. To our knowledge, this is the first work in which the relevance of the language of assessment in relation to affective symptoms has been assessed.

Finally, another important outcome of our study is that the Basque language translations of the DASS-42, PHQ-9, GAD-7 and PQ-B questionnaires have been validated. These tools are freely available, so that clinicians and researchers in the Basque region will be able to offer assessments in the individual’s preferred language.

## 5. Conclusions

The possible influence of the language in which the psychopathological examination is conducted, mainly in the expression of psychotic symptomatology, has been object of recurrent interest in recent years. Most of the studies carried out to date point to significant differences in the expression of psychotic symptoms when the examination is performed using the patient’s L1 vs. L2, and most of them point to a greater severity in L1. Interestingly, there are no studies published to date, neither using psychometric instruments as assessment measures, nor focused on affective symptomatology as main outcome. In our research, we have used a validation study of four questionnaires that measure psychotic, depressive and anxious symptomatology by means of self-reports, also collecting extensively the linguistic profile of the participants. Among the results, we have observed, firstly, that the higher the proficiency in the language of exploration, the milder the psychotic symptomatology detected, findings that contradict what has been described to date. On the other hand, and in a novel way, we have observed that L1 is consistently associated with greater severity in the depressive symptomatology detected, which is a first report related to affective symptomatology.

## Supporting information

S1 File Supplementary tables. (DOCX)
