# Supplementary material for: Reported severity of psychotic, depressive and anxiety symptoms in relation to bilingual language profile: An exploratory study and the validation of Basque versions of the PQ-B, DASS-42, PHQ-9 and GAD-7
Source: PLoS One. 2025 Mar 3;20(3):e0314069. doi: 10.1371/journal.pone.0314069 (PMC11875380; doi:10.1371/journal.pone.0314069)
Supplement: S1 File — (DOCX) [file pone.0314069.s001.docx]

Supplementary Table 1. Confirmatory analysis results of the assessed questionnaires.

|  | General group  (N=521) | Clinical group  (N=102) |
| --- | --- | --- |
| **DASS** |  |  |
| *Chi-square test of model fit* |  |  |
| Value | 1444.752 | 939.595 |
| DF | 816 | 816 |
| Value/DF | 1.77 | 1.15 |
| *RMSEA* |  |  |
| Estimate | 0.038 | 0.04 |
| 90% CI | 0.038 – 0.042 | 0.026-0.052 |
| *CFI/TLI* |  |  |
| CFI | 0.965 | 0.962 |
| 0.9TLI | 0.963 | 0.960 |
| **PQB** |  |  |
| *Chi-square test of model fit* |  |  |
| Value | 276.164 | 230.284 |
| DF | 189 | 189 |
| Value/DF | 1.46 | 1.22 |
| *RMSEA* |  |  |
| Estimate | 0.030 | 0.053 |
| 90% CI | 0.022 – 0.037 | 0.022-0.076 |
| *CFI/TLI* |  |  |
| CFI | 0.958 | 0.897 |
| TLI | 0.953 | 0.885 |
| **PHQ-9** |  |  |
| *Chi-square test of model fit* |  |  |
| Value | 58.765 | 36.268 |
| DF | 24 | 24 |
| Value/DF | 2.44 | 1.51 |
| *RMSEA* |  |  |
| Estimate | 0.053 | 0.072 |
| 90% CI | 0.036-0.07 | 0.0 – 0.117 |
| *CFI/TLI* |  |  |
| CFI | 0.986 | 0.980 |
| TLI | 0.979 | 0.970 |
| **GAD-7** |  |  |
| *Chi-square test of model fit* |  |  |
| Value | 27.945 | 24.082 |
| DF | 13 | 13 |
| Value/DF | 2.15 | 1.85 |
| *RMSEA* |  |  |
| Estimate | 0.047 | 0.091 |
| 90% CI | 0.022 – 0.071 | 0.028-0.148 |
| *CFI/TLI* |  |  |
| CFI | 0.993 | 0.970 |
| TLI | 0.989 | 0.951 |

DASS: Depression, Anxiety and Stress Scale; PQB: Prodromal questionnaire-Brief; PHQ-9: Patient Health Questionnaire-9; GAD-7: General Anxiety Disorder Scale-7; DF: Degrees of Freedom; RMSEA: Root Mean Square Error of Approximation; CFI: Comparative Fit Index; TLI: Tucker-Lewis Index

Supplementary Table 2. Reliability and concurrent validity

|  |  | Questionnaires | | | | | | | |
| --- | --- | --- | --- | --- | --- | --- | --- | --- | --- |
|  | DASS  Depression | | DASS  Stress | DASS  Anxiety | DASS  Total | PQB | PHQ-9 Factor 1 | PHQ-9  Factor 2 | GAD-7 |
| **General group (N=521)** |  | |  |  |  |  |  |  |  |
| DASS |  | |  |  |  |  |  |  |  |
| DASS Depression | **0.89** | |  |  |  |  |  |  |  |
| DASS Stress | 0.70 | | **0.90** |  |  |  |  |  |  |
| DASS Anxiety | 0.72 | | 0.73 | **0.89** |  |  |  |  |  |
| DASS Total | 0.91 | | 0.90 | 0.88 | **0.89** |  |  |  |  |
| PQB | 0.56 | | 0.57 | 0.61 | 0.64 | **0.81** |  |  |  |
| PHQ-9 |  | |  |  |  |  |  |  |  |
| PHQ Factor 1 | 0.77 | | 0.63 | 0.65 | 0.77 | 0.54 | **0.80** |  |  |
| PHQ Factor 2 | 0.60 | | 0.54 | 0.54 | 0.62 | 0.46 | 0.88 | **0.71** |  |
| GAD-7 | 0.62 | | 0.72 | 0.59 | 0.72 | 0.44 | 0.69 | 0.56 | **0.84** |
| **Clinical group (N=102)** |  | |  |  |  |  |  |  |  |
| DASS |  | |  |  |  |  |  |  |  |
| DASS Depression | **0.88** | |  |  |  |  |  |  |  |
| DASS Stress | 0.66 | | **0.90** |  |  |  |  |  |  |
| DASS Anxiety | 0.72 | | 0.76 | **0.89** |  |  |  |  |  |
| DASS Total | 0.90 | | 0.89 | 0.91 | **0.88** |  |  |  |  |
| PQB | 0.31 | | 0.33 | 0.33 | 0.36 | **0.83** |  |  |  |
| PHQ-9 |  | |  |  |  |  |  |  |  |
| PHQ Factor 1 | 0.80 | | 0.49 | 0.58 | 0.71 | 0.23 | **0.85** |  |  |
| PHQ Factor 2 | 0.52 | | 0.34 | 0.37 | 0.46 | 0.16 | 0.81 | **0.75** |  |
| GAD-7 | 0.73 | | 0.61 | 0.64 | 0.74 | 0.32 | 0.64 | 0.43 | **0.82** |

Bold numbers represent Cronbach’s alpha values. Alpha values > 0.70 show good reliability properties. DASS: Depression, Anxiety and Stress Scale;

PQB: Prodromal questionnaire-Brief; PHQ-9: Patient Health Questionnaire-9; GAD-7: General Anxiety Disorder Scale-7

Supplementary Table 3A. Factor loadings for Depression, Anxiety and Stress Scale (DASS)

|  | Factor loadings | | | | | |  |
| --- | --- | --- | --- | --- | --- | --- | --- |
|  | **General Group (N=521)** | | | **Clinical group (N=102)** | | |  |
|  | **Depression** | **Anxiety** | **Stress** | **Depression** | **Anxiety** | **Stress** |  |
| DASS-Depression items |  |  |  |  |  |  |  |
| 3 | 0.76 |  |  | 0.78 |  |  |  |
| 5 | 0.71 |  |  | 0.78 |  |  |  |
| 10 | 0.71 |  |  | 0.70 |  |  |  |
| 13 | 0.77 |  |  | 0.83 |  |  |  |
| 16 | 0.80 |  |  | 0.73 |  |  |  |
| 17 | 0.81 |  |  | 0.70 |  |  |  |
| 21 | 0.80 |  |  | 0.81 |  |  |  |
| 24 | 0.76 |  |  | 0.63 |  |  |  |
| 26 | 0.80 |  |  | 0.79 |  |  |  |
| 31 | 0.71 |  |  | 0.67 |  |  |  |
| 34 | 0.86 |  |  | 0.83 |  |  |  |
| 37 | 0.83 |  |  | 0.82 |  |  |  |
| 38 | 0.80 |  |  | 0.85 |  |  |  |
| 42 | 0.67 |  |  | 0.61 |  |  |  |
| DASS-Anxiety items |  |  |  |  |  |  |  |
| 2 |  | 0.43 |  |  | 0.49 |  |  |
| 4 |  | 0.60 |  |  | 0.55 |  |  |
| 7 |  | 0.57 |  |  | 0.72 |  |  |
| 9 |  | 0.63 |  |  | 0.55 |  |  |
| 15 |  | 0.58 |  |  | 0.42 |  |  |
| 19 |  | 0.39 |  |  | 0.53 |  |  |
| 20 |  | 0.70 |  |  | 0.63 |  |  |
| 23 |  | 0.43 |  |  | 0.30 |  |  |
| 25 |  | 0.60 |  |  | 0.62 |  |  |
| 28 |  | 0.76 |  |  | 0.64 |  |  |
| 30 |  | 0.63 |  |  | 0.75 |  |  |
| 36 |  | 0.68 |  |  | 0.64 |  |  |
| 40 |  | 0.68 |  |  | 0.68 |  |  |
| 41 |  | 0.63 |  |  | 0.73 |  |  |
| DASS-Stress items |  |  |  |  |  |  |  |
| 1 |  |  | 0.57 |  |  |  | 0.57 |
| 6 |  |  | 0.63 |  |  |  | 0.80 |
| 8 |  |  | 0.72 |  |  |  | 0.59 |
| 11 |  |  | 0.61 |  |  |  | 0.68 |
| 12 |  |  | 0.66 |  |  |  | 0.58 |
| 14 |  |  | 0.56 |  |  |  | 0.59 |
| 18 |  |  | 0.65 |  |  |  | 0.73 |
| 22 |  |  | 0.63 |  |  |  | 0.54 |
| 27 |  |  | 0.72 |  |  |  | 0.69 |
| 29 |  |  | 0.73 |  |  |  | 0.71 |
| 32 |  |  | 0.61 |  |  |  | 0.55 |
| 33 |  |  | 0.70 |  |  |  | 0.69 |
| 35 |  |  | 0.68 |  |  |  | 0.54 |
| 39 |  |  | 0.70 |  |  |  | 0.75 |

Supplementary Table 3B. Factor loadings for Prodromal Questionnaire-Brief (PQB)

|  | Factor loadings | |
| --- | --- | --- |
|  | **General group (N=521)** | **Clinical group** |
| **PQB items** |  |  |
| 1 | 0.45 | 0.33 |
| 2 | 0.33 | 0.55 |
| 3 | 0.27 | 0.48 |
| 4 | 0.28 | 0.52 |
| 5 | 0.50 | 0.32 |
| 6 | 0.53 | 0.20 |
| 7 | 0.35 | 0.48 |
| 8 | 0.52 | 0.33 |
| 9 | 0.28 | 0.50 |
| 10 | 0.45 | 0.53 |
| 11 | 0.37 | 0.65 |
| 12 | 0.50 | 0.36 |
| 13 | 0.38 | 0.43 |
| 14 | 0.47 | 0.39 |
| 15 | 0.45 | 0.52 |
| 16 | 0.39 | 0.47 |
| 17 | 0.44 | 0.61 |
| 18 | 0.52 | 0.49 |
| 19 | 0.29 | 0.56 |
| 20 | 0.30 | 0.60 |
| 21 | 0.57 | 0.19 |

Supplementary Table 3C. Factor loadings for Patient Health Questionnaire (PHQ)-9

|  | Factor loadings | |  |
| --- | --- | --- | --- |
|  | **General group (N=521)** | **Clinical group (N=102)** | |
| PHQ-9 items |  |  | |
| Factor 1 |  |  | |
| 1 | 0.73 | 0.85 | |
| 2 | 0.81 | 0.90 | |
| 6 | 0.70 | 0.77 | |
| 7 | 0.57 | 0.61 | |
| 8 | 0.47 | 0.49 | |
| 9 | 0.53 | 0.59 | |
| Factor 2 |  |  | |
| 3 | 0.92 | 0.83 | |
| 4 | 0.61 | 0.75 | |
| 5 | 0.60 | 0.69 | |

Covariance was specified between the error items of the following two pair of items: PHQ1 and PHQ2, and PHQ7 and PHQ8.

Supplementary Table 3D. Factor loadings for General Anxiety Disorder (GAD) Scale-7

|  | Factor loadings | |
| --- | --- | --- |
|  | **General group (N=520)** | **Clinical group (N=102)** |
| GAD-7 items |  |  |
| 1 | 0.77 | 0.79 |
| 2 | 0.79 | 0.73 |
| 3 | 0.71 | 0.66 |
| 4 | 0.72 | 0.60 |
| 5 | 0.62 | 0.56 |
| 6 | 0.47 | 0.53 |
| 7 | 0.52 | 0.50 |

Supplementary Table 4A. Stepwise regression : Prodromal Questionnaire-Brief (PQB) Total score. Analysis of Deviance after backward model selection with FDR-adjusted p-values.

| **General group** |  |  |  |  |  |
| --- | --- | --- | --- | --- | --- |
| PQB total ~ Exposure + Proficiency + L1 + Education + Gender +  Age + Exposure * Gender + L1 * Education  **AIC: 2272.1** |  | **Df** | **χ2** | **Pr(>χ2)** | **FDR adjusted p-value** |
| Exposure |  | 1 | 0.267 | 0.60541 | 0.63360 |
| Proficiency |  | 1 | 2.590 | 0.10757 | 0.15620 |
| L1 |  | 1 | 6.049 | 0.01392 | 0.06960 |
| Education |  | 2 | 13.761 | 0.00103 | **0.02190** |
| Gender |  | 1 | 0.764 | 0.38209 | 0.42990 |
| Age |  | 1 | 8.841 | 0.00295 | **0.02660** |
| Exposure * Gender |  | 1 | 2.294 | 0.12989 | 0.17710 |
| L1 * Education |  | 2 | 5.236 | 0.07294 | 0.14270 |
| **Clinical group** |  |  |  |  |  |
| PQB total ~ Exposure + Age + Exposure * Age  **AIC:** **385.22** |  |  |  |  |  |
| Exposure |  | 1 | 0.020 | 0.88760 | 0.89750 |
| Age |  | 1 | 5.222 | 0.02230 | 0.07800 |
| Exposure * Age |  | 1 | 3.519 | 0.06065 | 0.12010 |
| **Between-group analysis** |  |  |  |  |  |
| PQB total ~ group + AoA + Proficiency |  |  |  |  |  |
| Group |  | 1 | 13.965 | 0.00019 | **0.00080** |
| AoA |  | 1 | 3.710 | 0.05409 | 0.08530 |
| Proficiency |  | 1 | 7.536 | 0.00605 | **0.01810** |

AIC: Akaike Information Criterion; Df: degrees of freedom. Significant effects after FDR correction are highlighted in bold.

Supplementary Table 4B. Stepwise regression : Prodromal Questionnaire-Brief (PQB) Distress score. Analysis of Deviance after backward model selection with FDR-adjusted p-values.

| **General group** |  |  |  |  |  |
| --- | --- | --- | --- | --- | --- |
| distress ~ Education + Age  **AIC: 3142.9** |  | **Df** | **χ2** | **Pr(>χ2)** | **FDR adjusted p-value** |
| Education |  | 1 | 14.400 | 0.00075 | **0.02190** |
| Age |  | 1 | 3.532 | 0.06021 | 0.13960 |
| **Clinical group** |  |  |  |  |  |
| distress ~ 1  **AIC:** **532.32** |  |  |  |  |  |
| **Between-group analysis** |  |  |  |  |  |
| distress ~ Group + AoA + Proficiency + Exposure |  |  |  |  |  |
| Group |  | 1 | 14.498 | 0.00014 | **0.00070** |
| AoA |  | 1 | 5.547 | 0.01852 | **0.04070** |
| Proficiency |  | 1 | 8.601 | 0.00336 | **0.01110** |
| Exposure |  | 1 | 2.716 | 0.09933 | 0.14080 |

AIC: Akaike Information Criterion; Df: degrees of freedom. Significant effects after FDR correction are highlighted in bold.

Supplementary Table 5. Stepwise regression : Patient Health Questionnaire (PHQ)-9. Analysis of Deviance after backward model selection with FDR-adjusted p-values.

| **General group** |  |  |  |  |  |
| --- | --- | --- | --- | --- | --- |
| total ~ Exposure + Proficiency + L1 + Education + Age +  Proficiency *Age + Exposure * Age + L1*Education  **AIC: 2855.4** |  | **Df** | **χ2** | **Pr(>χ2)** | **FDR adjusted p-value** |
| Exposure |  | 1 | 1.064 | 0.30222 | 0.34870 |
| Proficiency |  | 1 | 4.819 | 0.02814 | 0.10740 |
| L1 |  | 1 | 4.096 | 0.04299 | 0.12090 |
| Education |  | 2 | 5.382 | 0.06781 | 0.13960 |
| Age |  | 1 | 1.572 | 0.20989 | 0.24860 |
| Proficiency * Age |  | 1 | 8.838 | 0.00295 | **0.02660** |
| Exposure * Age |  | 1 | 1.946 | 0.16298 | 0.21570 |
| L1 * Education |  | 2 | 4.150 | 0.12555 | 0.17660 |
| **Clinical group** |  |  |  |  |  |
| total ~ AoA + Exposure + Proficiency + L1 + Education + Gender + Age + AoA * Age + Proficiency * Gender + Exposure * Age + Exposure * Edu + L1*Gender  **AIC:** **630.24** |  |  |  |  |  |
| AoA |  | 1 | 11.552 | 0.00068 | **0.00940** |
| Exposure |  | 1 | 5.562 | 0.01836 | 0.07800 |
| Proficiency |  | 1 | 0.757 | 0.38438 | 0.48920 |
| L1 |  | 1 | 0.556 | 0.45574 | 0.55480 |
| Education |  | 3 | 7.305 | 0.06280 | 0.12010 |
| Gender |  | 1 | 0.075 | 0.78429 | 0.84460 |
| Age |  | 1 | 0.017 | 0.89746 | 0.89750 |
| AoA*Age |  | 1 | 3.589 | 0.05816 | 0.12010 |
| Proficiency * Gender |  | 1 | 2.182 | 0.13960 | 0.19540 |
| Exposure * Age |  | 1 | 3.639 | 0.05643 | 0.12010 |
| Exposure * Education |  | 3 | 8.584 | 0.03536 | 0.11000 |
| L1 * Gender |  | 1 | 2.327 | 0.12717 | 0.19540 |
| **Between-group analysis** |  |  |  |  |  |
| PHQ_total ~ group + AoA + Proficiency + Exposure +  L1 + Group * AoA + Group * Exposure + Group * L1  **AIC: 1142** |  |  |  |  |  |
| Group |  | 1 | 16.120 | 0.00006 | **0.00040** |
| AoA |  | 1 | 0.334 | 0.56339 | 0.56340 |
| Proficiency |  | 1 | 4.446 | 0.03499 | 0.06790 |
| Exposure |  | 1 | 2.481 | 0.11521 | 0.14080 |
| L1 |  | 1 | 9.120 | 0.00253 | **0.00930** |
| Group * AoA |  | 1 | 3.967 | 0.04641 | 0.08170 |
| Group * Exposure |  | 1 | 5.915 | 0.01501 | **0.03810** |
| Group * L1 |  | 1 | 6.305 | 0.01204 | **0.03310** |

AIC: Akaike Information Criterion; Df: degrees of freedom. Significant effects after FDR correction are highlighted in bold.

Supplementary Table 6. Stepwise regression: Generalized Anxiety Disorder Scale (GAD)-7. Analysis of Deviance after backward model selection with FDR-adjusted p-values.

| **General group** |  |  |  |  |  |
| --- | --- | --- | --- | --- | --- |
| total ~ AOA + Exposure + Proficiency + Gender + Age + AoA * Age + Proficiency * Age + Exposure * Age  **AIC: 2682.2** |  | **Df** | **χ2** | **Pr(>χ2)** | **FDR adjusted p-value** |
| AoA |  | 1 | 3.739 | 0.05315 | 0.13360 |
| Exposure |  | 1 | 0.101 | 0.75038 | 0.76740 |
| Proficiency |  | 1 | 3.730 | 0.05343 | 0.13360 |
| Gender |  | 1 | 4.346 | 0.03709 | 0.11770 |
| Age |  | 1 | 1.690 | 0.19361 | 0.23780 |
| AoA * Age |  | 1 | 2.706 | 0.10000 | 0.15620 |
| Proficiency * Age |  | 1 | 6.080 | 0.01367 | 0.06960 |
| Exposure * Age |  | 1 | 4.764 | 0.02906 | 0.10740 |
| **Clinical group** |  |  |  |  |  |
| total ~ Gender  **AIC: 577.6** |  |  |  |  |  |
| Gender |  | 1 | 3.540 | 0.05992 | 0.12010 |
| **Between-group analysis** |  |  |  |  |  |
| total ~ group  **AIC: 1057.3** |  |  |  |  |  |
| Group |  | 1 | 46.882 | <.001 | **<.001** |

AIC: Akaike Information Criterion; Df: degrees of freedom. Significant effects after FDR correction are highlighted in bold.

Supplementary Table 7A. Stepwise regression: Depression, Anxiety and Stress Scale (DASS) Total score. Analysis of Deviance after backward model selection with FDR-adjusted p-values.

| **General group** | | | | | |
| --- | --- | --- | --- | --- | --- |
| total ~ Proficiency + L1 + Education + Age + Proficiency*Age  **AIC: 3948.09** |  | **Df** | **χ2** | **Pr(>χ2)** | **FDR adjusted p-value** |
| Proficiency |  | 1 | 4.652 | 0.03102 | 0.10740 |
| L1 |  | 2 | 3.264 | 0.19553 | 0.23780 |
| Education |  | 1 | 0.516 | 0.47236 | 0.50610 |
| Age |  | 1 | 2.959 | 0.08539 | 0.14420 |
| Proficiency * Age |  | 2 | 4.511 | 0.10483 | 0.15620 |
| **Clinical group** | | | | | |
| total ~ AoA + L1 + Age + Education + AoA*Age  **AIC:** **851** |  |  |  |  |  |
| AoA |  | 1 | 7.970 | 0.00476 | **0.03330** |
| L1 |  | 1 | 2.640 | 0.10421 | 0.17160 |
| Age |  | 1 | 0.362 | 0.54720 | 0.61290 |
| Education |  | 3 | 6.180 | 0.10317 | 0.17160 |
| AoA * Age |  | 1 | 5.275 | 0.02163 | 0.07800 |
| **Between-group analysis** | | | | | |
| total ~ group + AoA + L1 + group*AoA + group*L1  **AIC: 1551.2** |  |  |  |  |  |
| Group |  | 1 | 23.387 | <.001 | **<.001** |
| AoA |  | 1 | 2.134 | 0.14404 | 0.15840 |
| L1 |  | 1 | 2.519 | 0.11251 | 0.14080 |
| Group * AoA |  | 1 | 2.549 | 0.11035 | 0.14080 |
| Group * L1 |  | 1 | 2.648 | 0.10369 | 0.14080 |

AIC: Akaike Information Criterion; Df: degrees of freedom. Significant effects after FDR correction are highlighted in bold.

Supplementary Table 7B. Stepwise regression: Depression, Anxiety and Stress Scale (DASS) Depression score. Analysis of Deviance after backward model selection with FDR-adjusted p-values.

| **General group** | | | | | |
| --- | --- | --- | --- | --- | --- |
| depression ~ Exposure + Proficiency + L1 + Age + Education + Proficiency * Age  **AIC: 2824.22** |  | **Df** | **Chi-square** | **Pr(>Chisq)** | **FDR adjusted p-value** |
| Exposure |  | 1 | 3.019 | 0.08228 | 0.14420 |
| Proficiency |  | 1 | 2.937 | 0.08655 | 0.14420 |
| L1 |  | 1 | 6.537 | 0.01056 | 0.06790 |
| Age |  | 1 | 0.035 | 0.85143 | 0.85140 |
| Education |  | 2 | 13.059 | 0.00146 | **0.02190** |
| Proficiency * Age |  | 1 | 3.372 | 0.06631 | 0.13960 |
| **Clinical group** | | | | | |
| depression ~ 1  **AIC: 704.09** |  |  |  |  |  |
| **Between-group analysis** | | | | | |
| depression ~ group + AoAz + Proficiency + Exposure +  L1 + Group * AoA + Group * Exposure + Group*L1 |  | **Df** | **Chi-square** | **Pr(>Chisq)** |  |
| Group |  | 1 | 15.772 | 0.00007 | **0.00040** |
| AoA |  | 1 | 3.705 | 0.05426 | 0.08530 |
| Proficiency |  | 1 | 2.376 | 0.12323 | 0.14520 |
| Exposure |  | 1 | 0.932 | 0.33429 | 0.34470 |
| L1 |  | 1 | 5.754 | 0.01645 | **0.03880** |
| Group * AoA |  | 1 | 3.945 | 0.04702 | 0.08170 |
| Group * Exposure |  | 1 | 2.632 | 0.10475 | 0.14080 |
| Group * L1 |  | 1 | 5.136 | 0.02343 | **0.04830** |

AIC: Akaike Information Criterion; Df: degrees of freedom. Significant effects after FDR correction are highlighted in bold.

Supplementary Table 7C. Stepwise regression: Depression, Anxiety and Stress Scale (DASS) Anxiety score. Analysis of Deviance after backward model selection with FDR-adjusted p-values.

| **General group** | | | | | |
| --- | --- | --- | --- | --- | --- |
| DASS anxiety ~ Education + Age  **AIC: 2600.1** |  | **Df** | **Chi-square** | **Pr(>Chisq)** | **FDR adjusted p-value** |
| Education |  | 2 | 10.296 | 0.00581 | **0.04360** |
| Age |  | 1 | 4.251 | 0.03923 | 0.11770 |
| **Clinical group** | | | | | |
| DASS anxiety ~ AoA + Exposure + Education + Age + AoA*Age+  Exposure * Age  **AIC: 646.25** |  |  |  |  |  |
| AoA |  | 1 | 10.819 | 0.00100 | **0.00940** |
| Exposure |  | 1 | 2.201 | 0.13792 | 0.19540 |
| Education |  | 3 | 7.250 | 0.06433 | 0.12010 |
| Age |  | 1 | 0.447 | 0.50357 | 0.58750 |
| AoA * Age |  | 1 | 6.656 | 0.00988 | 0.05530 |
| Exposure * Age |  | 1 | 2.072 | 0.15001 | 0.20000 |
| **Between-group analysis** | | | | | |
| DASS anxiety ~ group  **AIC: 1110.2** |  |  |  |  |  |
| Group |  | 1 | 82.043 | <.001 | **<.001** |

AIC: Akaike Information Criterion; Df: degrees of freedom. Significant effects after FDR correction are highlighted in bold.

Supplementary Table 7D. Stepwise regression: Depression, Anxiety and Stress Scale (DASS) Stress score. Analysis of Deviance after backward model selection with FDR-adjusted p-values.

| **General group** | | | | | |
| --- | --- | --- | --- | --- | --- |
| stress ~ Proficiency + L1 + Education + Age + Proficiency *Age + L1 * Education  **AIC: 3124.8** |  | **Df** | **Chi-square** | **Pr(>Chisq)** | **FDR adjusted p-value** |
| Proficiency |  | 1 | 3.325 | 0.06823 | 0.13960 |
| L1 |  | 1 | 4.652 | 0.03102 | 0.10740 |
| Education |  | 2 | 3.264 | 0.19553 | 0.23780 |
| Age |  | 1 | 0.516 | 0.47236 | 0.50610 |
| Proficiency * Age |  | 1 | 2.959 | 0.08539 | 0.14420 |
| L1 * Education |  | 2 | 4.511 | 0.10483 | 0.15620 |
| **Clinical group** | | | | | |
| Stress ~1  **AIC**: **670.98** |  |  |  |  |  |
| **Between-group analysis** | | | | | |
| Stress ~ group  **AIC: 1110.2** |  | **Df** | **Chi-square** | **Pr(>Chisq)** | **FDR adjusted p-value** |
| Group |  | 1 | 57.768 | 0.00000 | **<.001** |

AIC: Akaike Information Criterion; Df: degrees of freedom. Significant effects after FDR correction are highlighted in bold.

Supplementary Material 8 – Language Proficiency Questionnaire

**LANGUAGE PROFICIENCY QUESTIONNAIRE**

Please provide us with some basic information about your knowledge and use of the different languages you use. As this information is especially relevant to us, please take the time to answer each question.

1. List the languages you know according to your **proficiency level**, i.e. rank them according to your general **degree of knowledge** (your native language should be first).

1___________________ (from now on Lang [1])

2___________________ (from now on Lang [2])

3___________________ (from now on Lang [3])

4___________________ (from now on Lang [4])

1. Please provide the average percentage of time you are **exposed** to each of the languages, i.e. the percentage of time you read, listen, write and speak in each of the languages (the sum of the percentages must add up to 100%):

| **Language** | **Percentage** |
| --- | --- |
| Lang [1] |  |
| Lang [2] |  |
| Lang [3] |  |
| Lang [4] |  |
| Sum | 100 % |

1. Indicate the age (in years) at which you started learning:

Lang [1] ______ Lang [2] ______ Lang [3] ______ Lang [4] ______

1. Have you ever lived in a country where Spanish and Basque were not the official languages? If so, indicate the number of months and the language spoken in that country (if more than one country, indicate the languages and the months in each):

|  | **Months** |
| --- | --- |
| Language(s) _____________________ |  |

1. Indicate the level of proficiency in Language [1] on a scale from 0 to 10 (10 is the highest score and corresponds to the highest level of proficiency) in...

|  | **Lang [1]** | **Lang [2]** | **Lang [3]** | **Lang [4]** |
| --- | --- | --- | --- | --- |
| **…speaking** |  |  |  |  |
| **…understanding** |  |  |  |  |
| **…writing** |  |  |  |  |
| **…reading** |  |  |  |  |

1. Do you have any **official qualification** in any language you know? (If the answer is YES, please specify)

**Lang [1] Lang [2] Lang [3] Lang [4]**

YES ______ YES ______ YES ______ YES ______

NO NO NO NO

1. If you had to lose all the languages you know except one, which language would you choose to keep?

_________________________________

1. What is/are the mother tongue(s) of...

your father? ________________________

your mother? ________________________

1. Which hand do you write with?________________________
